# Does job stress mediate the risk of work disability due to common mental disorders among social workers compared with other health and social care, education, and non-human service professionals? A prospective cohort study of public sector employees in Finland

**DOI:** 10.5271/sjweh.4171

**Published:** 2024-09-01

**Authors:** Otso Rantonen, Jenni Ervasti, Kristina Alexanderson, Tuula Oksanen, Ville Aalto, Ellenor Mittendorfer-Rutz, Paula Salo

**Affiliations:** 1 Department of Psychology and Speech-Language Pathology, University of Turku, Turku, Finland.; 2 Finnish Institute of Occupational Health, Helsinki, Finland.; 3 Department of Clinical Neuroscience, Division of Insurance Medicine, Karolinska Institutet, SE-171 77 Stockholm, Sweden.; 4 Institute of Public Health and Clinical Nutrition, University of Eastern Finland, Kuopio, Finland.

**Keywords:** counterfactual mediation analysis, human service profession, sick leave, sickness absence, job strain, effort-reward imbalance

## Abstract

**Objective:**

This study aimed to investigate (i) the risk of work disability (>10-day sickness absence spell or disability pension) due to common mental disorders (CMD) among social workers compared with other health and social care, education, and non-human service professionals and (ii) whether the risk was mediated by job stress.

**Methods:**

A cohort of 16 306 public sector professionals in Finland was followed using survey data from baseline (2004 or if not available, 2008) on job stress [job strain or effort-reward imbalance (ERI)] and register data on work disability due to CMD from baseline through 2011. A Cox proportional hazards model was used to analyze the risk of work disability due to CMD between three occupation-pairs in a counterfactual setting, controlling for age, sex, job contract, body mass index, alcohol risk use, smoking, and physical inactivity.

**Results:**

Social workers’ job stress was at higher level only when compared to education professionals. Thus, the mediation hypothesis was analyzed comparing social workers to education professionals. Social workers had a higher risk of work disability due to CMD compared with education professionals [hazard ratio (HR) 2.08, 95% confidence interval (CI) 1.58–2.74]. This HR was partly mediated by job strain (24%) and ERI (12%). Social workers had a higher risk of work disability than non-human service professionals (HR 1.54, 95% CI 1.13–2.09), but not compared with other health and social care professionals.

**Conclusions:**

Job stress partly mediated the excess risk of work disability among social workers only in comparison with education professionals.

Human service professionals (eg, nursing professionals, preschool teachers, eldercare professionals) have a high risk of work disability (sickness absence and/or disability pension) due to common mental disorders (CMD), and employment in social work has been associated with a particularly high risk (1–5). CMD refer to the two most prevalent mental disorders – depressive and anxiety disorders – that are characterized by negative impacts of the mood or feelings of the individual (6). In the working population, the risks of CMD (7–9), sickness absence (4), and disability pension (10, 11) have been associated with job strain (12) and effort-reward imbalance (ERI) (13), which are arguably the two most prominent models for work-related stress. Job strain is defined as high job demands combined with low job control, and ERI describes a situation where employees put more efforts into their work than what they receive as intrinsic or extrinsic rewards (12, 13). High job demands (eg, workload and pace of work) (14–18) and ERI (19) have been observed in social work. However, job control may conversely be high in social work due to autonomy in decision making (15). Thus, it is unclear whether the stressful combination of high job demands and low control (ie, job strain) is higher in social work than other human service professions.

Some previous studies have investigated the mediating effect of job stress for sickness absence among human service professionals (20, 21). No mediating effect of job strain among health and social care professionals compared with other female employees (21) or ERI among human service professionals compared with non-human service professionals (20) was observed. However, a limitation of those studies was that they used the traditional difference method for mediation analysis (22), which does not consider potential interaction between exposure and mediator. This is an unrealistic assumption in most studies and may result in invalid inferences (23). In this prospective cohort study of public sector employees in Finland, we applied the counterfactual framework, which enables mediation analysis in the presence of exposure–mediator interactions and nonlinearities. Also, in this framework, the total effect (TE) of the exposure and the mediator on the outcome can be decomposed into the natural direct effect (NDE) and the natural indirect effect (NIE), which improves statistical inferences (23, 24). Our aim was to investigate (i) social workers’ risk of work disability due to CMD is higher than that of other social and healthcare professionals, education professionals, and non-human service workers and (ii) whether job strain and ERI mediate those associations.

## Methods

### Subjects and data collection

A prospective cohort study was conducted. We linked questionnaire data from the Finnish Public Sector Study (25) with register data from the Social Insurance Institution of Finland and the Finnish Centre for Pensions on work disability (regarding sickness absence spells >10 days or disability pensions) due to CMD (26). The cohort comprised working-aged employees (18–63 years) from ten Finnish municipalities. The employees were sent questionnaires in 2004 (N=32 322, response rate 65%) and 2008 (N=38 727, response rate 70%). We included social workers, and other health and social care, education, and non-human service professionals (office workers and secretaries), respectively, who had responded to the 2004 and/or the 2008 survey.

For those who responded to both surveys, we used their answers in the 2004 survey. This led to 10 304 (63%) respondents from the 2004 survey and 6002 (37%) respondents from the 2008 survey, in all 16 306 respondents. For respondents in 2004, the mean follow-up time was 2182.6 (SD 716.6) days. For respondents in 2008, the mean follow-up time was 1029.6 (SD 207.8) days. Inclusion criteria were having ≥6 months of employment based on employers’ registers, no disability pension or old-age pension (part- or full-time), no ongoing sickness absence spell ≥90 days or antidepressant treatment (>30 defined daily dosages) (27) during the survey year and being alive at baseline on 1 January after the survey response.

### Exposure

We used occupation-pairs as the exposure variable. Social workers were first paired with other health and social care professionals; secondly with education professionals; and thirdly with non-human service professionals. Occupation was defined according to the occupational classification by Statistics Finland (28), which is based on the ISCO-88 classification (International Classification of Occupations) (supplementary appendix https://www.sjweh.fi/article/4171). ISCO-88 codes were derived at baseline from employers’ registers.

### Outcome

We derived data on sickness absence spells that were ≥11 days long and disability pensions (temporary, permanent, and full- and part-time) with CMD ICD-10 diagnoses: depressive (F30–F39) or anxiety disorders (F40–F48) (26) and calculated work disability spells due to CMD by combining overlapping and consecutive spells, using the diagnosis of the first period. Data on sickness absence spells were obtained from a register kept by Social Insurance Institution of Finland, who handles reimbursements for sickness absence spells >10 days (the first day is a waiting day, days 2–10 are reimbursed by the employer). The Social Insurance Institution may pay sickness absence benefits for up to 300 days during two consecutive years. After that period, the employee has to apply for temporary or permanent disability pension or return to work. Data on disability pensions were obtained from the Finnish Centre for Pensions. Follow-up for work disability spells due to CMD began on 1 January the year after the survey response and ended at the first occurrence of the studied outcome (sickness absence or disability pension due to CMD), old-age pension, death, or end of the register data availability at 31 December 2011. Mean follow-up was 4.8 (SD 2.2) years.

### Potential mediators

Studied mediator variables were job strain (12) and ERI (13). Questionnaire and item descriptions have been presented in previous studies (10, 29). Job control was measured with nine items for job control (scale 1=low to 5=high control). Job demands was measured with three items for job demands (ie, workload and pace of work) (scale 1=low to 5=high demands). Job strain was calculated by subtracting the mean sum score of job control from the mean sum score of job demands thus producing a measure ranging from -4 to 4. A negative score indicated low job strain (eg, higher control than demands).

Efforts put into work were measured with one item for the amount of experienced effort at work (scale 1=low to 5=high efforts). Rewards received from work were measured with three items for income and job benefits, recognition and prestige, and personal satisfaction from work (scale 1=low to 5=high rewards). ERI was measured by dividing the response for the item measuring efforts at work by the mean subscale score rewards at work thus producing a measure ranging from 0.2 to 5. A high score indicated higher ERI (eg, high efforts relative to rewards). Job strain and ERI were treated as continuous in the analyses. In addition, we conducted a sensitivity analyses with dichotomous job strain and ERI, which is presented in supplementary appendix.

### Covariates

We included register-based data from the Finnish Public Sector Study on age (continuous), sex (male/female), job contract (temporary/permanent), and multiple self-reported lifestyle factors as binary variables (30). These were body mass index (BMI, <25 kg/m^2^=not overweight, >25 kg/m^2^=overweight), high-risk alcohol consumption (>288 g/week for men and >192 g/week for women=yes, below high-risk use=no), current smoking (yes/no), and recommended level of physical activity [>14 metabolic equivalent task (MET) hours/week=yes, below recommended MET=no].

### Statistical analysis

First, we investigated whether social workers and the other professionals differed regarding sociodemographic, health, and job stress at baseline. Group differences were tested with χ^2^-test for dichotomous variables, and with t-test for continuous variables.

The risk of work disability due to CMD across occupations was analyzed with Cox proportional hazards regression. The proportional hazards assumption was confirmed based on the Wald χ2-test (P>0.05) and visual inspection of the Kaplan–Meier curves (supplementary appendix). We examined the association between occupation-pairs (social work versus other health and social care professionals; social work versus education professionals; social work versus non-human service work) and risk of future work disability due to CMD, using the Cox proportional hazards model, controlling for age, sex, job contract, BMI, alcohol risk use, smoking, and physical inactivity.

We hypothesized that social workers had higher job strain and ERI than the reference professions, and investigated mediation only when this was the case, following guidelines of mediation analysis (23, 24). Then we proceeded with mediation analysis only in cases where our hypothesis about a higher job stress was true. TE was presented in all cases. For mediation analysis, we used counterfactual mediation analysis with survival data, calculating hazard ratios (HR) with 95% confidence intervals (CI). Counterfactual mediation analysis with survival data has been described elsewhere in detail (31).We conducted the analyses using the SAS macro presented by Valeri & VanderWeele (23, 24), allowing for interaction even when the interaction was not significant, following the guidelines for counterfactual analysis (32).

Five assumptions were made in the analyses (23, 24, 31): (i) no unmeasured exposure-outcome confounding; (ii) no unmeasured mediator-outcome confounding; (iii) no unmeasured exposure-mediator confounding; (iv) no mediator-outcome confounder affected by the exposure; and (v) temporal ordering of being employed in the profession and a self-rated level of the mediator was assumed in that order, in that the level of mediator was caused by exposure to the occupation. Mediation was examined by decomposing the total effects into controlled direct effect (CDE), NDE, NIE, and TE. Their calculations are presented below (23). The CDE refers to the HR for the association between employment in social work (exposure A=1) compared with employment in one of the reference occupations (A=0), and work disability due to CMD, when setting job strain/ERI (mediator=M) to a set level (M=m) uniformly over the study population. This resembles a scenario where the effect of employment in social work is not mediated through job strain or ERI.

CDE=Hazard (A=1, M=m) / Hazard (A=0, M=m)

The NDE is calculated by holding the value of the mediator in social work to the same level with the reference profession, ie, a scenario where job strain or ERI are at a similar level to the reference profession in social work (M_A=0_). In the presence of no interaction between exposure and mediator, CDE and NDE are equivalent.

NDE=Hazard (A=1, M_A=0_) / Hazard (A=0, M_A=0_)

The NIE refers to the excess risk of work disability due to CMD among social workers that is solely due to their higher job strain or ERI.

NIE=Hazard (A=1, M_A=1_) / Hazard (A=1, M_A=0_)

In TE, both NDE and NIE are summed up and thus considered to estimate the association between being employed in social work and work disability due to CMD. The TE represents an interpretable population average over the levels of the mediator, even in the presence of exposure-mediator interaction, and the TE estimate depends on the prevalence of the mediator in the study population (32).

TE=NDE × NIE

Dissecting the NIE of social workers on risk of work disability due to CMD allowed us to estimate the extent to which the excess risk of work disability among social workers would be reduced if their job strain or ERI was at the same level as in the reference occupation.

1 / NIE=Hazard (A=1, M_A=0_) / Hazard (A=1, M_A=1_)

We used SAS 9.4 Statistical Package for all analyses (SAS Institute Inc, Cary, NC, USA).

## Results

The cohort comprised 16 306 individuals, of which 535 (3%) were social workers (table 1a). Most of the participants were women (87%) and > 40 years old (68%). Average age was similar among social workers and other human service professionals (43–44 years), but among non-human service professionals it was a few years higher (48 years). During the follow-up, 15% of social workers had at least one work disability spell due to CMD; this proportion was higher than the incidence among education professionals (7%) and non-human service professionals (11%), and similar to that among other health and social care professionals (14%) (table 1a). Descriptive statistics for dichotomous covariates and the outcome are presented in tables 1a and 1b.

**Table 1a:** Frequency and percentages for dichotomous variables by occupation. [N=frequency; SW=social workers; HSC=health and social care professionals; EDU=education professionals; Non-H=non-human service professionals; ref=reference; CMD=common mental disorders]. Scales and variable definitions are shown in supplementary appendix.

Dichotomous variables		SW N=535 (3.3%)				Other HSC N=5711 (35.0%)			SW vs HSC		EDU N=7441 (45.6%)			SW vs EDU		Non-H N=2619 (16.1%)			SW vs Non-H		All N=16 306 (100.0%)	
		N	%	ref		N	%		P-value		N	%		P-value		N	%		P-value		N	%
**Covariates**																						
	Women	472	88.2	ref		5383	94.3		<0.0001		5958	80.1		<0.0001		2319	88.6		0.83		14 132	86.7
	Type of job contract (permanent)	395	74.1	ref		5054	88.5		<0.0001		5970	80.2		<0.001		233	89.1		<0.0001		13 752	84.4
	Current smoking (yes)	72	13.8	ref		1087	19.5		<0.01		625	8.5		<0.0001		623	24.4		<0.0001		2407	15.0
	Alcohol consumption (high risk)	62	11.6	ref		423	7.4		<0.001		752	10.2		0.28		252	9.7		17		1489	9.2
	Low physical activity	106	20.0	ref		1293	22.8		0.13		1546	21.0		0.58		788	30.3		<0.0001		3733	23.1
	Under- or overweight	207	39.7	ref		2524	45.6		<0.01		2650	36.5		0.14		1170	46.5		<0.01		6551	41.4
**Outcome (cases)**																						
	Work disability due to CMD	81	15.1	ref		806	14.1		0.52		527	7.1		<0.0001		284	10.8		<0.01		1698	10.4

**Table 1b:** Means and standard deviation (SD) for continuous variables by occupation.

Continuous variables		SW				Other HSC			SW vs HSC		EDU			SW vs EDU		Non-H			SW vs Non-H		All	
		Mean	SD	ref		Mean	SD		P-value		Mean	SD		P-value		Mean	SD		P-value		Mean	SD
Covariates																						
	Age	44.3	10.0	ref		44.2	9.8		0.82		43.4	9.5		0.04		47.6	9.1		<0.001		44.3	9.7
Mediators																						
	Job strain	-0.4	1.0	ref		-0.3	1.0		0.60		-0.7	0.9		<0.001		-0.1	1.0		<0.001		-0.4	1.0
	ERI	1.6	0.5	ref		1.6	0.5		0.29		1.5	0.5		<0.001		1.7	0.7		<0.05		1.5	0.5

Contrary to our hypotheses, differences in job strain and ERI were relatively small between the professions. Job stress was similar among social workers (job strain -0.4, ERI 1.6) compared with other health and social care professionals (job strain -0.3, ERI 1.6), and even slightly lower than among non-human service professionals (job strain -0.1, ERI 1.7). However, in line with our mediation hypothesis, job strain and ERI were somewhat higher among social workers than among education professionals (job strain -0.7, ERI 1.5) (table 2.). The sample range for job strain and ERI was the same as the theoretical range (job strain: -4.0–4.0; ERI: 0.25–5.0).

**Table 2:** Counterfactual mediation analysis on the association between ‘social work versus education’ and work disability due to mental disorders with job strain and effort–reward imbalance (ERI) as a mediators with exposure-mediator interaction allowed. [HR=hazard ratio; CI=confidence interval.]

		HR ^a^	95% CI	P for interaction	Proportion mediated (%)
Job strain as mediator				0.08	
	Controlled direct effect	1.99	1.55–2.55		
	Natural direct effect	1.82	1.37–2.41		
	Natural indirect effect	1.14	1.05–1.24		
	Total effect	2.08	1.58–2.74		24
ERI as mediator				0.36	
	Controlled direct effect	1.48	0.75–2.96		
	Natural direct effect	1.96	1.51–2.54		
	Natural indirect effect	1.06	1.02–1.11		
	Total effect	2.09	1.61–2.69		12

^a^ Adjusted for sex, age, job contract, smoking, alcohol use, body mass index, and physical activity.

### Social workers compared to education professionals

In the model with job strain as mediator, being a social worker was associated with higher risk of work disability compared to education professionals (HR 1.99, 95% CI 1.55–2.55) (ie, the CDE). In a scenario in which job strain among social workers was at the same level as that among education professionals (ie, the NDE), the HR for being a social worker was slightly lower: 1.82 (95% CI 1.37–2.41). However, as social workers had slightly higher job strain than education professionals (-0.4 versus -0.7), the association with CMD-related work disability due to higher job strain (ie, the NIE), was 1.14 (95% CI 1.05–1.24). Thus, theoretically, the excess risk of work disability due to CMD would decrease by 14% if social workers’ job strain would be decreased to the same level than that of education professionals. Multiplying the NDE and NIE of job strain led to a HR of 2.08 (95% CI 1.58–2.74) (ie, TE). Job strain mediated 24% of the association between being a social worker and work disability due to CMD (table 3.).

In the model with ERI as a mediator, being a social worker was associated with higher risk of work disability compared to education professionals (HR 1.48, 95% CI 0.75–2.96), that is, being a social workers was not associated with the risk of work disability due to CMD in the adjusted model. In a scenario in which ERI among social workers was at the same level as that among education professionals, being a social worker was associated with the higher risk (HR 1.96, 95% CI 1.51–2.54). However, as social workers had slightly higher ERI than education professionals (1.6 versus 1.5, respectively), the risk of CMD-related work disability due to higher ERI was 1.06 (95% CI 1.02–1.11) times higher solely because of the higher ERI. This indicates a 6% reduction in the excess risk of work disability due to CMD among social workers in a scenario, where ERI would be at the same level than observed in education professionals. Multiplying the NDE and NIE of job strain led to a HR of 2.09 (95% CI 1.61–2.69). ERI mediated 12% of the association between being a social worker and work disability due to CMD (table 3).

### Social workers compared to non-human service professionals

When both the NDE and NIE were combined (TE), being a social worker was associated with a higher risk of work disability due to CMD compared to non-human service professionals (HR 1.54, 95% CI 1.13–2.09) when accounting for all covariates and job strain. The risk estimate remained same when replacing job strain with ERI (HR 1.54, 95% CI 1.13–2.12) (not reported in tables). As the means of job strain and ERI were lower among social workers compared to non-human service professionals, our mediation hypothesis was rejected.

### Social workers compared to other health and social care professionals

When both the NDE and NIE were combined (TE), being a social worker was not associated with a higher risk of work disability due to CMD compared to other health and social care professionals when accounting for all covariates and job strain (HR 1.18, 95% CI 0.87–2.59), or when accounting for covariates and ERI (HR 1.15, 95% CI 0.88–1.51) (not reported in tables). Without exposure–outcome association, there was no point to study mediation.

### Sensitivity analyses

Social workers were more likely to have high job strain than education professionals and had the highest percentage for ERI of all professions. However, in sensitivity analyses, the HR were in general similar, but slightly smaller (see supplementary appendix). Although a higher percentage of social workers had high job strain than education professionals, the risk of CMD-related work disability due to high versus low job strain (ie, the NIE), was only 1.04 (95% CI 0.98–1.09). In the other comparisons, with ERI as a mediator, the small HR indicated little mediation. The TE for employment in social work in these comparisons were similar to the main analyses.

## Discussion

In this prospective cohort study of public sector employees in Finland, we found that the excess in risk of work disability due to CMD among social workers compared with education professionals was partly explained by higher job stress, as indicated by job strain and ERI. Social workers had about 2-fold higher risk for future work disability due to CMD compared with education professionals, 1.5-fold higher risk compared with non-human service professionals, but no excess risk compared with other health and social care professionals.

The results from mediation analysis indicated that theoretically, the excess risk of future work disability due to CMD would decrease by 14% for job strain and 6% for ERI if social workers job stress would decrease to the level observed in education professionals. As for other occupational group comparisons, despite having a higher risk of work disability due to CMD, social workers had lower levels of job stress thus rejecting our hypothesis of job stress as the mediator. The risk of work disability did not differ between social workers and other health and social care professionals. In our sensitivity analyses, the results showed that differences between occupations in job stress were small and thus with more general dichotomous measures, these differences decreased and the HR were smaller, indicating little mediation in any comparison (see supplemenary appendix).

Job strain and ERI have been identified as major risk factors for CMD (7, 8), sickness absence (4) and disability pension (10, 11). Thus, it was plausible to assume that these indicators of job stress would mediate the excess risk of CMD previously observed in social workers. We found that job stress mediated the excess risk of work disability due to CMD among social workers compared with education professionals, but the mediation hypotheses did not hold when compared with other health and social care professionals, nor non-human service professionals.

Compared with education professionals, job stress explained 12–24% of the excess risk of work disability due to CMD. The NDE indicates the association between employment in social work (including the psychosocial conditions or other risk factors associated with the occupation) with the outcome, through pathways that don’t involve job stress (ie, a scenario where job stress was at a similar level with the reference profession). In our study, this was almost 2-fold with either mediator in comparison with education professionals. Thus, probably also other work-related factors or selection into or out of profession play a major role for the excess risk. Indeed, previous studies have shown that social workers, particularly child welfare professionals, encounter several work-related psychosocial risk factors, such as client work demands, high quantitative and learning demands, role conflicts and moral distress due to lack of resources (14–18, 33).

Similarly to previous studies (5), employment in social work was associated with a higher risk of work disability due to CMD compared with employment in non-human service professions. However, in contrast with previous findings (19), in our study ERI and job strain was higher among non-human service professionals. To our knowledge, studies have not compared job strain between social workers and other professions. Thus, other factors likely mediate the excess risk. Also, unmeasured exposure–outcome confounding could have impacted the results.

Previous studies indicate that client work factors are potential mediators for the association between employment in social work and work disability due to CMD (20, 21). One study (20) found that emotional demands, risk of threats and violence, and low work time control mediated the association between employment in human service professions compared with non-human service professions and a higher risk of self-reported sickness absence. High efforts, low rewards, or low social support did not mediate the association. Another study (21) found that job strain had no impact on the risk of sickness absence (≥ 21 days) among female health and social care professionals in comparison to other female employees (including non-human service professionals as a major group), and instead emotional demands and risk of threats and violence mediated the risk. Such client work conditions may be particularly demanding in social work compared with other human service professions, which could explain the higher risk of work disability due to CMD in social work (34–36).

Comparison of social workers with other health and social care professionals showed no difference in the risk of work disability due to CMD. In general, most health and social service professionals have an elevated risk of CMD (1, 2). Especially health and social care professionals with low socioeconomic status (eg, eldercare professionals) are more likely to report high psychosocial demands (eg, high client work demands and low job resources) (37, 38). Future studies could further investigate which factors mediate the risk of CMD among specific health and social care professionals. In addition, a selection of employees, who have a higher risk of experiencing job stress or developing mental disorders, into these professions may be a mediator (18, 35). However, it is not clear whether this is more likely in social work compared with other human service professions. Also, a recent study showed that the association between emotional demands and depression was not accounted for by pre-employment depression or reporting bias (ie, experiencing higher emotional demands due to previous or current mental disorders) (35). This indicates that the association between employment in social work (and the work exposures that come with the job characteristics) and work disability due to CMD is not fully accounted by pre-employment mental disorders.

### Strengths and limitations

Strengths of this study include a large sample, a relatively long follow-up (3–7 years), and the combination of survey and register data. We used high-quality administrative register data on sickness absence and disability pension, which provided a valid measure of work disability due to CMD without recall bias or common-method bias, and no drop-out during follow-up (39, 40). For the exposure variables, we used survey data from two well-established measures of job stress, which have been validated in multiple previous studies. The survey had a high response rate that ensured good generalizability. We analyzed the data using the counterfactual framework, allowing for detailed investigation of NDE and NIE.

There were also limitations in our study. The sample in our study was limited to public sector employees. In Finland, however, most social workers and teachers are employed in the public sector and thus, the sample should be quite representative regarding those occupations, but not necessarily regarding non-human service professionals. The follow-up ended in 2011 due to data availability and thus the results provide only an estimate for the current situation. In social work, the high risk of work disability due to CMD has likely remained or even elevated because psychosocial conditions have deteriorated. Studies show that social workers report high job demands and high moral distress due to lack of resources. Further, poor economic conditions have likely increased the needs among clients in marginalized populations (16, 33). Sex-stratified analysis was not possible due to too few male social workers in the sample. A recent study showed higher hazards of antidepressant treatment among male social workers compared to female employees in comparisons with non-human service professionals (5). Thus, mediation by job stress may also be different for male and female social workers compared with other professionals, which could be further investigated in larger samples. All in all, our sample of social workers was relatively small.

The outcome measure of work disability was rather crude. Our data did not include short sickness absence spells (1–10 days), nor did we differentiate between the incidences of long-term sickness absence, shorter spells (ie, ≥11 days sickness absence spell) or disability pensions in our data. Based on statistics by the Social Insurance Institution of Finland and the Finnish Centre For Pensions (41), we calculated that among all employees in Finland about 13–14% of the new work disability spells due to mental disorders (F00–F99) in 2004, 2008 and 2011 were disability pensions. However, in the public sector the incidence of disability pensions, particularly of part-time disability pensions, is higher than in private sector (42). Thus, we approximate that in our data, about 15–20% of the 1698 work disability episodes due to mental disorders were disability pensions.

Future studies should try to obtain data that separates sickness absence from disability pension and examine different work disability lengths. Moreover, if an employee had a sickness absence spell or disability pension due to another diagnosis than CMD, that person was at that time not at risk for sickness absence or disability pension due to CMD before returning to work. A previous study among social workers showed that social workers had relatively similar rates of work disability due to somatic disorders compared with employees in two teaching professions, and psychologists. Thus, the bias caused by such competing risks is likely small at least for the comparisons between social workers and education professionals.

Unmeasured confounding could have impacted the results. For example, pre-employment mental disorders were not measured and such conditions could impact the level of exposure (higher selection of such employees to social work), mediator (vulnerability to job stress) and outcome (higher risk of work disability due to CMD). However, it is unlikely that they fully account for the association (35). Moreover, we argue that the relatively large sample size in our study increases the randomization of individuals to occupations and decreases the selection impact due to a more balanced distribution of such selection bias between the professions. This reduces the impact of the background risk caused by a selection bias, due to higher exchangeability of the background risks between the individuals in the exposure groups (eg, social work versus education professionals) (43). Still, there may be a systematic selection of individuals that could impact the results, for example by overestimating the impact of employment in social work on the risk of CMD, or the impact of job stress on CMD. Other potential confounders could for example be physical work environment, biological or social factors. Confounders should be considered in future studies.

Lower socioeconomic status (SES) is associated with elevated risk of mental disorders (44). Although professions within most occupational groups in the current study had a similar level of education, and thus SES, there are some differences in educational level especially in the group of health and social care professions (eg, nurses versus doctors). However, we were unable to adjust for differing within-occupational group educational level in the analyses. This may have caused minor overestimation to the estimates.

Job strain and ERI are general measures of job stress, and we did not measure occupation-specific risk factors in social work that may be associated with the high risk of work disability due to CMD. However, a low sensitivity of measures for different groups is a natural feature of a comparative study across occupations that is based on measuring common exposure variables and their impact. A wide range of other work-related risk factors in social work have been identified (14–18), which are in general related to aspects such as poor job design, not being valued and respected at the workplace, and occupational uncertainty, and these have been associated with the risk of work disability due to CMD (9). Occupational differences may also be explained by accumulation of multiple risk factors (37) or quality of job demands (eg, type of clientele, emotional demands or risk of threats or violence) in contrast to quantitative demands (34). Future studies could investigate other risk factors prevalent in social work that were not measured in this study (15, 17, 18), preferably with counterfactual mediation analysis, compared with traditional mediation analysis.

### Implications

Our study has two major implications. First, health and social care professionals and social workers in particular have an elevated risk of work disability due to CMD. Second, our findings suggest that balancing job demands and control as well as the high efforts and low rewards in social work could reduce the risk of work disability due to CMD to some extent, but that other factors are likely more important. Previous studies suggest that potential mediators are likely related to high client work demands. In addition to those, a major challenge in social work is the high rate of turnover and lack of qualified employees. Reducing turnover would also likely reduce job strain and ERI as well as client work demands. Policy makers could reduce job stress related to lack of resources and turnover in social work by allocating adequate resources into social work (16–18, 33), but the psychosocial conditions should be improved as well.

Identification of potential risk factors is important, because it can guide organizations in planning interventions and preventive strategies to counter the high risk of work disability due to CMD and support return to work. For example, organizational and professional commitment, job satisfaction, a supportive organizational culture and climate, reduced workload, peer and management support and supervision have been associated with decreased burnout and turnover and higher employee resilience for high job demands (18). Thus, organizational practices that develop these aspects could also reduce the risk of work disability due to CMD. Future studies could utilize randomized controlled trials to investigate whether such practices could buffer the risk of work disability due to CMD in social work. Also, research is needed to provide information about effective organizational interventions for reducing job strain and ERI.

### Concluding remarks

Social workers had a higher risk of work disability due to CMD as compared with education and non-human service professionals. The risk was similar between social workers and other health and social care professionals. Job stress mediated the excess risk of work disability only in comparison with education professionals and only to a rather small extent.

## Supplementary material

Supplementary material
