# Supplementary material for: Does job stress mediate the risk of work disability due to common mental disorders among social workers compared with other health and social care, education, and non-human service professionals? A prospective cohort study of public sector employees in Finland
Source: Scand J Work Environ Health. 2024 Aug 30;50(6):456–65. doi: 10.5271/sjweh.4171 (PMC11393659; doi:10.5271/sjweh.4171)
Supplement: Supplementary material [file SJWEH-50-456-S001.pdf]

Does job stress mediate the risk of work disability due to common mental disorders among social workers compared with other health and social care, education, and non-human service professionals? A prospective cohort study of public sector employees in Finland[1]

by Otso Rantonen,[2] Jenni Ervasti, Kristina Alexanderson, Tuula Oksanen, Ville Aalto, Ellenor Mittendorfer-Rutz, Paula Salo

1. Supplementary material
2. Correspondence to: Otso Rantonen, University of Turku, Department of Psychology, University of Turku, Assistentinkatu 7 (Publicum building), 20014 Turku, Finland. [E-mail: optran@utu.fi]

Appendix. Cohort description, proportionality assumption tests and sensitivity analyses

Occupational classifications

We selected all human service occupations that were available in the Finnish Public Sector Study and office workers as a non-human service profession. Selection criteria was having the relevant occupational code in 2005 according to the occupational classifications. The occupational categories were based on the Classification of Occupations 2001 created by Statistics Finland. The codes are based on the ISCO-88 (International Classification of Occupations) with minor changes (1). Based on previous studies we formed four major occupational categories from the occupations (2–5). These were social workers, other health and social care professionals, education professionals and non-human service professionals (Table S1).

Screening for social workers within the employees with ISCO code for social work

In addition, we reviewed the employers' registers and selected employees from organizations that provide social work. This was done by an experienced social worker and researcher, who did not have other connections to this study.

Turnover from occupation during follow-up

We had no annual data for occupation was not available, so job turnover was not taken into account.

Inclusion criteria at baseline (1st January after the survey response)

We included employees who at baseline 1) had responded to the survey in 2004, or secondarily in 2008, in the Finnish Public Sector Study (FPS), 2) were employed in the selected professions (ISCO code), 3) had employment for at least 6 months (employer records), 4) were alive, 5) not retired, 7) were healthy based on register data on sickness absence and antidepressant treatment, 8) were 18–63 years old at baseline and had availability for antidepressant data. Further specification is presented in Table S2.

Table S1. Occupational sectors, occupations and ISCO-88 codes

Sector	Occupation	ISCO-88 code
	Social workers	2446
**Other health & social care professionals**	Medical doctors	2221
	Psychologists	2445
	Physiotherapists	3226
	Nursing and midwifery professionals and associate professionals	223, 323 51321, 51322, 51324
	Social work associate professionals	3460
	Home-based Personal care workers	5133
**Education professionals**	Secondary education teaching professionals	231, 232
	Primary Education Teaching professionals	2331
	Pre-primary education teaching professionals	2332
	Special education teaching professionals	2340
**Non-human service professionals**	Administrative and executive secretaries, and General, keyboard and customer clerks	41, 42

Table S2. Cohort selection with inclusion criteria

Inclusion criteria at baseline (1st January after the survey response)	Description
**Response to survey in 2004, or secondarily in 2008, in the Finnish Public Sector Study (FPS)**	Responses from the 2004 questionnaire were used if available. If no response in 2004, then data from 2008 were used. In 2004, 32,322 employees responded. In the whole FPS sample the response rate was 65%. In 2008 38,727 employees responded. In the whole FPS sample the response rate was 70%. In total, the cohort in our study was 16, 306 employees.
**Occupation at baseline (see Table S1.)**	ISCO-88 code. Additionally, we reviewed the employer's registers and selected employees, who were employed in organizations that provide social work services
**Employed in the selected occupation after baseline for at least 6 months**	At least 6 months of employment. Occupations were defined based on the employer's records
**Alive**	No deaths at baseline
**Not retired**	No old-age pension at baseline
**Health status**	During the questionnaire response year (2004 or 2008), no disability pension, no active sickness absence spells lasting more than 365 days and no antidepressant treatment (DDD ≥ 30), as well as no active sickness absence spell lasting (≥ 90 days) at baseline.
**Age**	18–63 years at baseline
**Data availability for antidepressants**	The municipality of Tampere was excluded in analyses of future antidepressants, due to no data availability.

Proportionality assumption and Kaplan-Meier curves

The Wald $\chi2$-test was used to test the proportionality assumption. The test showed support for the proportionality assumption for work disability due to CMD ($p > .05$). The Kaplan-Meier curve is presented in Figure S1. The curves were assessed visually by plotting the log-log Kaplan Meier survival estimates against the log of time and evaluating whether the curves are relatively parallel. Visual assessment of the Kaplan-Meier curves showed support for the proportionality assumption.

Figure S1. Kaplan Meier curves for the odds of work disability due to CMD among social workers compared with social and health care, education and non-human service professionals

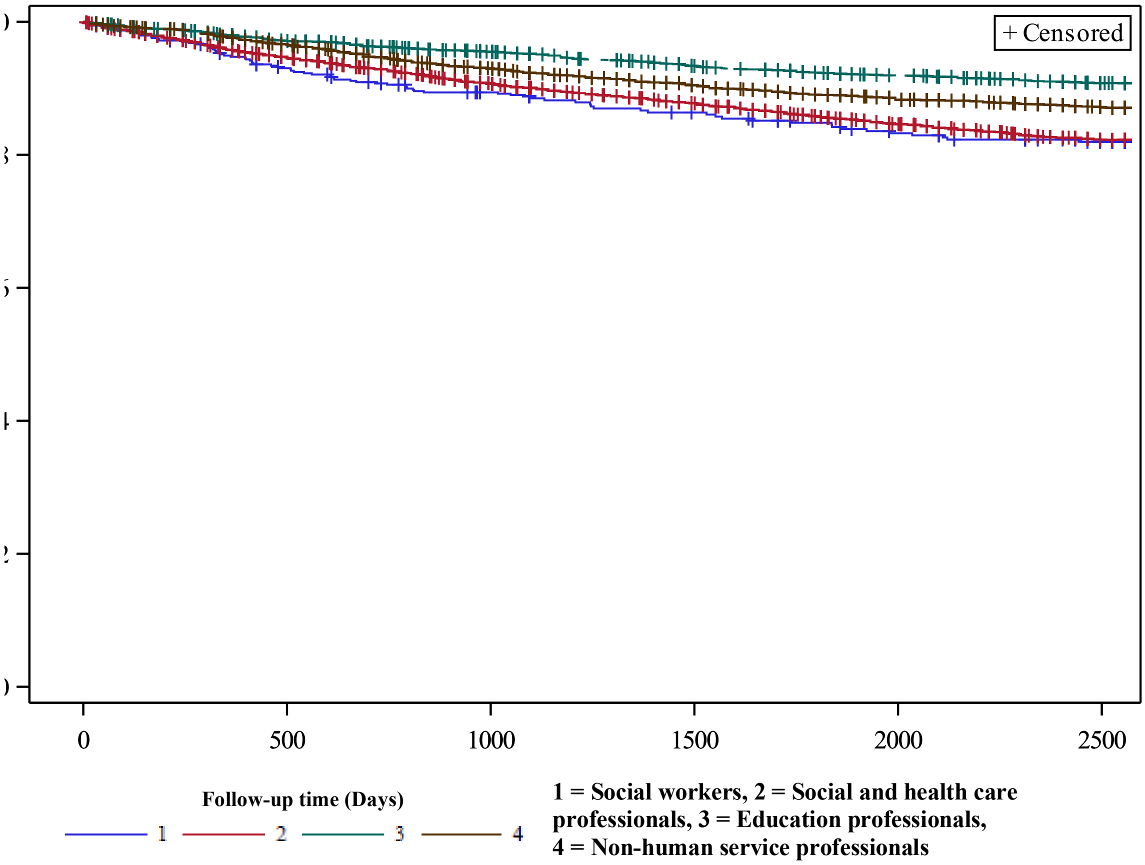

Sensitivity analyses with dichotomous job strain and ERI

For sensitivity analyses, both high job strain and high ERI were dichotomized based on 2010–2014 medians (2010–2012 for ERI) in the longitudinal Finnish Public Sector Study for job demands (>3.4) and control (<3.777), and medians for efforts (>4.0) and rewards (<3). If both job demands and control, or both efforts and rewards indicated adverse psychosocial conditions based on the median, we specified the individuals with high job strain and effort-reward imbalance.

A higher percentage of social workers had high job strain compared with education professionals (17% vs 10%, $p < .001$), and the percentage was lower compared with health and social care (24%) and non-human service professionals (26%). The percentage of ERI among social workers (18%) was higher compared with education (13%, $p < .05$), health and social care (15%, $p < .05$) and non-human service professionals (17%, $p > .05$). Thus we proceeded with the mediation analysis for job strain only compared with education professionals, and with ERI in all comparisons. The HRs for each effect (CDE, NDE, NIE and TE) with job strain as mediator are presented in table S3, and the effects with ERI are presented in table S4.

Social workers compared to education professionals

In the model with dichotomous job strain as mediator, the adjusted HR for social workers compared to education professionals was 2.02 (95% CI 1.53–2.65) (i.e., the CDE). In a scenario in which job strain among social workers was at the same level as that among education professionals (i.e., the NDE), the HR for social workers was 1.99 (95% CI 1.55–2.54), i.e. at the same level to the CDE due to no interaction ($p>.05$). Although, a higher percentage of social workers had high job strain than education professionals, the risk of CMD-related work disability due to high vs. low job strain (i.e., the NIE), was only 1.04 (95% CI 0.98–1.09). Thus, theoretically, the excess risk of work disability due to CMD would decrease by 4% if the same amount of social workers had high job strain compared with education professionals. Multiplying the direct and indirect effects of job

strain led to a HR of 2.06 (95% CI 1.61–2.62) (i.e., TE). The proportion of the risk of work disability due to CMD mediated by job strain was 7%.

In the model with dichotomous ERI as mediator, the adjusted HR for social workers compared to education professionals was 1.98 (95% CI 1.49–2.62) (i.e., the CDE). In a scenario in ERI among social workers was at the same level as that among education professionals (i.e., the NDE), the HR for social workers was 2.04 (95% CI 1.60–2.60), i.e. at the same level to the CDE due to no interaction (p>.05). Although, a higher percentage of social workers had high ERI than education professionals, the risk of CMD-related work disability due to high vs. low ERI (i.e., the NIE), was only 1.03 (95% CI 0.99–1.07). Thus, theoretically, the excess risk of work disability due to CMD would decrease by 3% if the same amount of social workers had high ERI compared with education professionals. Multiplying the direct and indirect effects of ERI led to a HR of 2.10 (95% CI 1.65–2.68) (i.e., TE). The proportion of the risk of work disability due to CMD mediated by ERI was 5%.

Social workers compared to non-human service professionals

When accounting for high job strain, when both the natural direct effect and indirect effect were combined (TE), social had a higher risk of work disability due to CMD compared to non-human service professionals (TE: HR = 1.50, 95% CI 1.16–1.95). As the percentage of high job strain was lower among social workers compared to non-human service professionals and the percentage of ERI similar, our mediation hypothesis was rejected.

In the model with dichotomous ERI as mediator, employment in social work was associated with a higher risk of work disability due to CMD compared to non-human service professionals (HR: 1.48, 95% CI 1.09–2.00), when adjusting for high job strain (i.e., the CDE). In a scenario in which ERI among social workers was at the same level as that among non-human service professionals (i.e., the NDE), the association remained and HR was similar to the CDE due to no interaction (HR: 1.47, 95% CI 1.13–1.92). Although, a higher percentage of social workers had high ERI than non-human service professionals, the association of employment in social work and risk of CMD-related work

disability due to high vs. low job strain (i.e., the NIE), was only 1.02 (95% CI 0.99–1.05). Thus, theoretically, the excess risk of work disability due to CMD would decrease by 2% if the same amount of social workers had high job strain compared with non-human service professionals. Multiplying the direct and indirect effects of job strain led to a HR of 1.50 (95% CI 1.15–1.95) (i.e., TE). The proportion of the risk of work disability due to CMD mediated by job strain was 6%.

Social workers compared to other health and social care professionals

When both the natural direct effect and indirect effect were combined (TE), employment in social work was not associated with a higher risk of work disability due to CMD compared to other health and social care professionals, when accounting for high job strain (TE: HR = 1.13, 95% CI 0.89–1.44). Without exposure-outcome association, there was no point to study mediation.

In the model with dichotomous ERI as mediator, the adjusted HR for social workers did not indicate an association for a higher risk of work disability due to CMD compared to other health and social care professionals (HR: 1.05, 95% CI 1.53–2.65) (i.e., the CDE). In a scenario in which ERI among social workers was at the same level as that among health and social care professionals (i.e., the NDE), the HR for social workers was 1.11 (95% CI 1.55–2.54), indicating no association. Although, a higher percentage of social workers had high ERI than other health and social care professionals, the association of employment in social work and risk of CMD-related work disability due to high vs. low job strain (i.e., the NIE), was only 1.03 (95% CI 0.99–1.07). Thus, theoretically, the excess risk of work disability due to CMD would decrease by 3% if the same amount of social workers had high job strain compared with other health and social care professionals. Multiplying the direct and indirect effects of job strain led to a HR of 1.14 (95% CI 0.90–1.45) (i.e., TE). The proportion of the risk of work disability due to CMD mediated by job strain was 22%, although the NIE was small.

Table S3. Counterfactual mediation analysis on the association between ‘social work vs education’, and work disability due to mental disorders with dichotomous effort-reward imbalance as mediators with exposure-mediator interaction allowed

Job strain as mediator	HR*	95 % CI	P for interaction	Proportion mediated (%)
**Social work vs education (ref.):**			0.74	
Controlled direct effect	2.02	1.53–2.65		
Natural direct effect	1.99	1.55–2.54		
Natural indirect effect	1.04	0.98–1.09		
Total effect	2.06	1.61–2.62		7%

*Adjusted for sex, age, job contract, smoking, alcohol use, BMI, and physical activity

Table S4. Counterfactual mediation analysis on the association between social work vs health and social care, education and non-human service professions and work disability due to mental disorders with dichotomous effort-reward imbalance as mediators with exposure-mediator interaction allowed

Effort-reward imbalance as mediator	HR*	95 % CI	P for interaction	Proportion mediated (%)
**Social workers vs. other health and social care professionals (ref.):**			0.23	
Controlled direct effect	1.05	0.80–1.38		
Natural direct effect	1.11	0.87–1.41		
Natural indirect effect	1.03	0.99–1.07		
Total effect	1.14	0.90–1.45		22%
**Social workers vs. education professionals (ref.):**			0.58	
Controlled direct effect	1.98	1.49–2.62		
Natural direct effect	2.04	1.60–2.60		
Natural indirect effect	1.03	0.99–1.07		
Total effect	2.10	1.65–2.68		5%
**Social workers vs. Non-human service professionals (ref.):**			0.95	
Controlled direct effect	1.48	1.09–2.00		
Natural direct effect	1.47	1.13–1.92		
Natural indirect effect	1.02	0.99–1.05		
Total effect	1.50	1.15–1.95		6%

*Adjusted for sex, age, job contract, smoking, alcohol use, BMI, and physical activity

References

1. Finland S. Classification of Occupations 2001. Helsinki: Statistics Finland; 2001. Available at: https://www.stat.fi/en/luokitukset/ammatti/ammatti_1_20010101/. (cited 27.01.2024).
2. Buscariolli A, Kouvonen A, Kokkinen L, Halonen JI, Koskinen A, Väänänen A. Human service work, gender and antidepressant use: a nationwide register-based 19-year follow-up of 752 683 women and men. Occup Environ Med. 2018;75(6):401–406. https://doi.org/10.1136/oemed-2017–104803.
3. Heinonen N, Lallukka T, Lahti J, Pietiläinen O, Nordquist H, Mänty M, et al. Working Conditions and Long-Term Sickness Absence Due to Mental Disorders: A Prospective Record Linkage Cohort Study Among 19- to 39-Year-Old Female Municipal Employees. J Occup Environ Med. 2022;64(2). https://doi.org/10.1097/JOM.0000000000002421.
4. Madsen IEH. Person-related work and incident use of antidepressants: relations and mediating factors from the Danish work environment cohort study. Scand J Work Environ Health. 2010;36(6):435. https://doi.org/10.5271/sjweh.3049.
5. Rantonen O, Alexanderson K, Pentti J, Kjeldgård L, Hämäläinen J, Mittendorfer-Rutz E, et al. Trends in work disability with mental diagnoses among social workers in Finland and Sweden in 2005–2012. Epidemiol Psychiatr Sci. 2017;26(6):644–654. https://doi.org/10.1017/S2045796016000597.
